# Gluteal Compartment Syndrome After Femoral Nail Extraction: A Case Report

**DOI:** 10.7759/cureus.37289

**Published:** 2023-04-08

**Authors:** Yosuke Kuroki, Ryuta Imamura, Hayato Inoue, Takahiro Inoue, Toshihiro Ebihara, Kimitaka Nakamura, Teiyu Izumi, Takahiro Hamada, Akihiko Inokuchi, Takeshi Arizono

**Affiliations:** 1 Orthopedic Surgery, Kyushu Central Hospital of the Mutual Aid Association of Public School Teachers, Fukuoka, JPN

**Keywords:** conservative treatment, body mass index, nail extraction, femoral shaft fracture, gluteal compartment syndrome

## Abstract

Gluteal compartment syndrome is a rare disorder and no definitive treatment has yet been established. Fasciotomy is often the treatment of choice for gluteal compartment syndrome, but there have been only a few cases that have improved with conservative therapy. A 26-year-old male with a body mass index of 40.5 who underwent femoral nail extraction surgery had severe pain in the right buttock and numbness in the right lower extremity. Initially, we suspected transient pain due to prolonged exposure to the same posture, but muscle weakness in the lower extremities and worsening of renal function appeared over time. Orthopedic evaluation revealed physical examination findings and MRI imaging findings consistent with gluteal compartment syndrome. Conservative treatment with temporary dialysis was chosen instead of fasciotomy because of the time required for diagnosis. Dialysis was started on postoperative day 3, renal function and muscle weakness recovered over time, and the patient was discharged home on postoperative day 37. At six months post-op, the patient was walking without pain and he had no changes in his peripheral neurologic examination compared to his preoperative baseline. Orthopedic surgeons should always be aware of the possibility of gluteal compartment syndrome when especially obese patients with prolonged operation times appeal to acute buttock pain. Diagnosis should be made as early as possible to get a good prognosis.

## Introduction

Acute compartment syndrome (CS) is an orthopedic emergency that causes ischemic injury to muscles and nerves due to decreased capillary perfusion pressure in the compartment caused by increased pressure in the compartment surrounded by fascia [1]. In many cases, the condition is precipitated by trauma [1,2]. Acute CS is potentially fatal if the diagnosis is delayed. However, it is often overlooked. Gluteal CS (GCS) has been reported as a rare form of acute CS. In this report, we describe a case of GCS that developed following nail extraction after surgery for a femoral shaft fracture that was treated successfully by conservative measures.

## Case presentation

The patient was a 26-year-old man with a body mass index (BMI) of 40.5 who sustained a left femoral shaft fracture and a left lateral tibial plateau fracture when he skidded and collided with a car after braking suddenly to avoid a right-turning car while riding a motorcycle and hit the left side of the body severely. Osteosynthesis was performed on the second day after the injury. There was no particular family history or psychosocial history.

**Figure 1:**
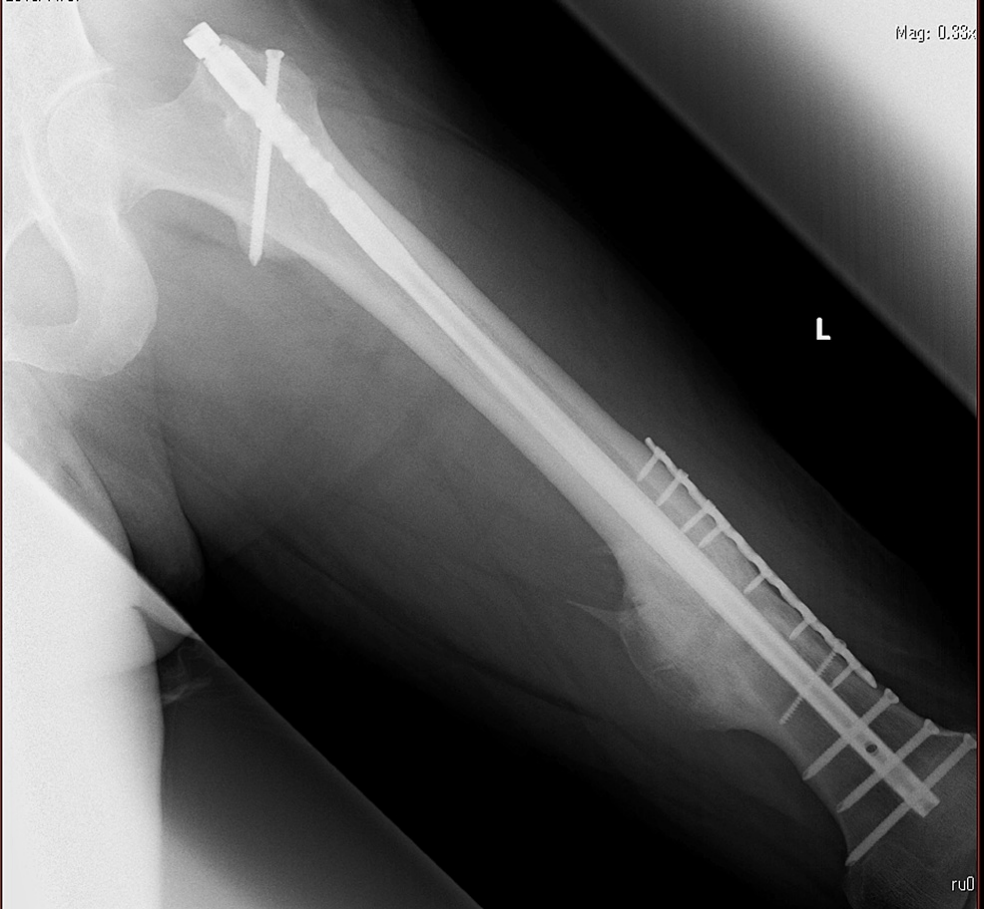
Left femur preoperative X-ray Bone fusion has been achieved.

The patient developed pseudoarthrosis postoperatively, for which he underwent surgery two years later. Four years after the surgery, he was admitted to our department for nail extraction surgery because bone fusion had been achieved (Figure 1).

**Figure 2:**
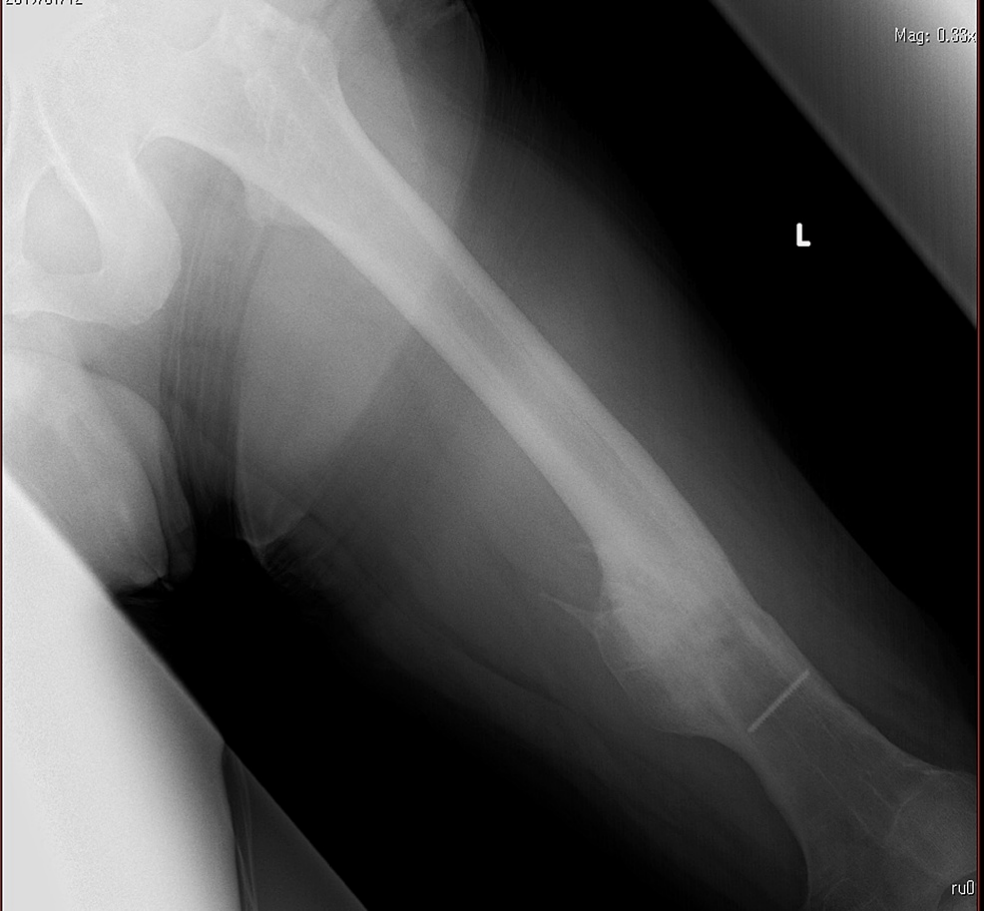
Left femur postoperative X-ray No abnormalities

The surgery was performed under general anesthesia on a traction operating table with the lower extremity in extension and the uninvolved side in hip flexion and external rotation. Because of the post-operative pseudoarthroplasty, bone proliferation was significant and manipulation was difficult. We attempted to remove the nail after removing the screw for the lateral stop, but the surgical field was so deep that the nail could not be removed easily and it took a long time. The postoperative X-ray showed no unusual initial findings (Figure 2). The operation time was four hours and eight minutes and the anesthesia time was five hours and 29 minutes. Blood loss was 562 mL.

After awakening from anesthesia, the patient complained of severe pain in the right buttock and numbness in the right lower extremity. At that time, his manual muscle test (MMT) score was 5 in both lower extremities.

Initially, we suspected transient pain as a result of prolonged exposure to the same position. However, on the morning of the first postoperative day, the right buttock muscle tissue was observed to be rigid. The MMT had decreased to 4 in the right lower extremity overall and to 1 for dorsiflexion of the right ankle joint. Thereafter, the MMT results improved over time. However, blood tests showed creatine kinase (CK) level of 72,985 U/L (normal range 0-170 U/L), blood urea nitrogen (BUN) level of 46.9 mg/dL (normal range 8−20 mg/dL), creatinine level of 5.97 mg/dL (baseline 1.05mg/dL), and hemoglobin of 12.0 g/dL (normal range 13.0-16.6 g/dL). A diagnosis of rhabdomyolysis and GCS was made based on the markedly high CK level and acute worsening renal function. Blood tests on postoperative day two showed metabolic acidosis and electrolyte abnormalities with potassium (K) of 4.9 mmol/L, calcium (Ca) of 7.1 mg/dL (normal range 8.4-10.4 mg/dL), pH of 7.274, bicarbonate (HCO3^-^) of 20.6 mEq/L (normal range 22-28 mEq/L). We decided on a conservative treatment plan because it took several days to diagnose GCS.

**Figure 3:**
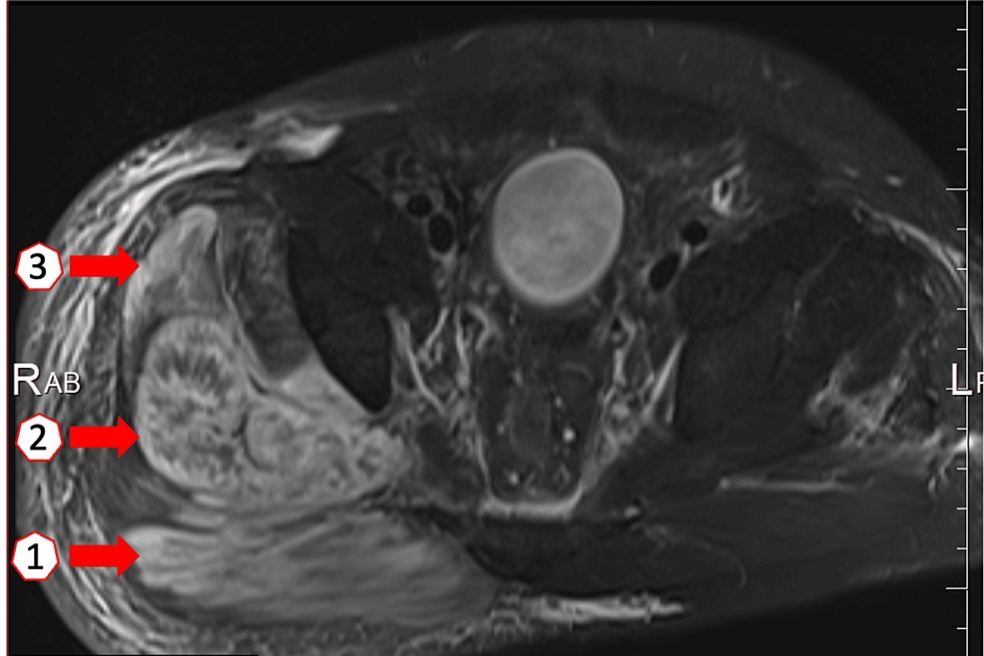
A transverse image of magnetic resonance scan (Short Tau Inversion Recovery) Edema and swelling of the gluteus maximus (①), gluteus medius (②), and gluteus minimus (③) muscle

The oliguria persisted, and blood tests on postoperative day four showed worsening renal function with BUN of 85.5 mg/dL, creatinine of 10.85 mg/dL, and hemoglobin of 8.5 g/dL. We considered the anemia to be caused by decreased production of erythropoietin due to decreased renal function and dilution due to massive transfusions, and performed blood transfusions as appropriate. The patient was started on dialysis three times a week. A magnetic resonance scan showed edematous signal changes in gluteal muscles (Figure 3).

On postoperative day 19, blood tests showed that the CK had improved to 255 U/L, BUN to 25.8 mg/dL, and creatinine to 1.80 mg/dL. Therefore, dialysis was terminated. On postoperative day 36, the BUN and creatinine values had improved to 11.4 mg/dL and 0.95 mg/dL, respectively, and the patient was discharged home on postoperative day 37.

Thereafter, the patient continued with outpatient follow-up. At two months postoperatively, although numbness remained beyond the right ankle joint, the pain had disappeared and he returned to work. Six months after the surgery, the numbness had disappeared. He has remained pain-free in the three years since surgery.

## Discussion

GCS is a rare disease with no established treatment, although 139 cases have been reported. In a meta-analysis by Adib et al., surgery was the most common cause of GCS (41%), followed by drug abuse or loss of consciousness (35%) and trauma (19%) [3]. Known risk factors for the development of GCS related to surgery include operation time (and its extension), patient size, use of epidural anesthesia, and intraoperative positioning [3]. The mortality rate of GCS is about 9%, but it is a serious disease with long-term neurological deficits in about 41% of cases [3].

Our patient was obese with a body weight of 140 kg and a BMI of 40.5 and an operation time that was longer than originally planned, so the risk of GCS was high. Although there are no clear diagnostic criteria for GCS, it is generally diagnosed by clinical symptoms. The most common symptoms include severe buttock pain, swelling and edema of the buttocks, erythema, and decreased sensation and muscle weakness in the area innervated by the sciatic nerve. Complications such as rhabdomyolysis and acute renal failure are known to occur secondary to compartment syndrome. Anatomically, the buttock compartment is divided into three major anatomical components, namely, gluteus maximus, gluteus medius/gluteus minimus, and tensor fascia femoris. Although the sciatic nerve does not travel within the components of this compartment, it is susceptible to compression because of its proximity [4,5]. An objective diagnostic indicator is the compartment pressure measured in the buttock. Although there is much debate regarding pressure measurement, most authors consider a compartment pressure greater than 30 mmHg to be an indication for fasciotomy [6]. However, this value is disputed, and some authors recommend performing fasciotomy when the difference between the intracompartmental pressure and diastolic pressure is less than 30 mmHg [7]. 

When GCS is diagnosed using these clinical symptoms and objective measures, and symptoms of nerve deficits such as lower extremity muscle weakness are present, fasciotomy is the treatment of choice. Adib et al. reported that about 80% of patients in their case series had undergone fasciotomy [3]. The remaining patients (approximately 20%) were treated with medical therapy, such as dialysis. There was no statistically significant difference in mortality between patients who were treated medically and those who were treated surgically. Although there were more posttreatment neurological deficits in the group that was treated medically, there was no between-group difference in the patients who initially had no neurological deficit [3]. On the other hand, some authors found increased morbidity and mortality in patients who underwent myotomy more than 24 hours after diagnosis of compartment syndrome because of increase in the risk of infection [8-10]. In the absence of a neurologic deficit, conservative treatment without fasciotomy may be indicated after consultation with the patient. Adib et al. also suggests that medical management with monitoring of neurological function may be the optimal first treatment for patients who initially have no neurological deficit [3]. In this case, it had been more than 24 hours since the surgery, and the patient was deemed to be at high risk of infection and other complications from the myotomy. Furthermore, given that his muscle weakness had improved over several hours, we decided to pursue conservative medical treatment in view of the risks and benefits of fasciotomy [8-11]. Ultimately, the patient had a good course without permanent neurological abnormalities, although temporary dialysis was performed for acute renal failure.

Intraoperative repositioning may be effective for relief of prolonged intraoperative pressure and to promote restoration of blood flow in severely obese patients when the operation time is prolonged [7], as in the present case. For the patient, his body position during surgery was slightly elevated on the affected side while the unaffected side was relatively low, which may have resulted in pressure being concentrated on the buttocks. It may have been possible to promote reperfusion in our case by rotating the traction bed to the affected or unaffected side for a few minutes every few hours. However, the present case was a simple nail removal procedure, and we did not anticipate a prolonged operation or GCS.

In the literature, the most common surgical cause of buttock CS is orthopedic surgery. Therefore, orthopedic surgeons should always assume rhabdomyolysis-CS in patients with a high BMI, even with routine scheduled surgery, and pay attention to intraoperative positioning and operation time.

## Conclusions

In this report, we describe a case of GCS that occurred after nail extraction and improved without sequelae after medical treatment. Orthopedic surgeons should always be aware of the possibility of GCS when operating on patients with a high BMI, regardless of whether the surgery is scheduled or an emergency. The diagnosis should be made as early as possible to get good prognosis.
